# Assessment of maternal and perinatal death surveillance and response (MPDSR) implementation in health facilities in Kigoma, Tanzania: a descriptive cross-sectional study

**DOI:** 10.1186/s12884-026-08775-1

**Published:** 2026-02-12

**Authors:** Patricia Spencer, Sifang Kathy Zhao, Abdulaziz Msuya, Alicia Ruiz, Sarah Huber-Krum, Sunday Dominico, Ahmad Makuwani, Florina Serbanescu

**Affiliations:** 1 Present Address: Division of Reproductive Health, U.S. Centers for Disease Control and Prevention, Atlanta, USA; 2 National Foundation for the Centers for Disease Control and Prevention, Atlanta, USA; 3 Thamini Uhai, Dar es Salaam, Tanzania; 4 Division of Reproductive, Maternal and Child Health, Ministry of Health, Dar es Salaam, Tanzania

**Keywords:** Maternal and perinatal death surveillance and response (MPDSR), Implementation readiness, Health facility assessment, Sustainable practice

## Abstract

**Background:**

Maternal mortality remains a critical public health concern in many countries. To reduce maternal mortality, the Tanzanian Ministry of Health is committed to implementing Maternal and Perinatal Death Surveillance and Response (MPDSR). We assessed the level of MPDSR system readiness in hospitals and health centers in a remote region in Tanzania and examined facility characteristics that may influence MPDSR sustainability.

**Methods:**

A cross-sectional study design was used to assess MPDSR implementation readiness in 11 hospitals and 29 health centers in the Kigoma region using a Health Facility Assessment in 2023. Data were abstracted from each facility to inform the MPDSR implementation readiness phase (i.e., early adoption, evidence of practice, integrated practice, or sustainable practice). Fisher’s exact test was used to test the associations between facility characteristics and facilities with sustainable practice.

**Results:**

The MPDSR implementation readiness in hospitals and health centers varied: 25% were in the early adoption phase, 20% in the evidence of practice phase, 32.5% in the integrated practice phase, and 22.5% in the sustainable practice phase. Sustainable MPDSR practice (*n =* 9) was associated with urban facility location (*p* = 0.0014), not having a staffing gap for midwife/nurse officer/enrolled nurse (*p* = 0.0072), higher facility delivery volume (*p* = 0.0472), higher proportion of deliveries with direct obstetric complications (*p* = 0.0036), and having personnel trained in MPDSR (*p* = 0.0051).

**Conclusion:**

Implementation of the MPDSR in Kigoma region has been challenging, with less than one in four of the assessed facilities demonstrating sustainable practice. Large, urban health facilities with an adequate number of skilled staff who were trained in MPDSR and provided care to a high volume of obstetric complications were most likely to have reached sustainable practice.

**Clinical trial number:**

Not applicable.

**Supplementary Information:**

The online version contains supplementary material available at 10.1186/s12884-026-08775-1.

## Background

Maternal mortality remains a critical public health concern across sub-Saharan Africa, accounting for approximately 70% of maternal deaths globally in 2023 [1]. The United Republic of Tanzania remains among the countries with the highest number of maternal deaths, ranking 7th in the world in 2023 [1], an increase from its 2020 level [2]. Causes of death include direct obstetric complications (84%) (e.g., obstetric hemorrhage, eclampsia, maternal sepsis), and indirect obstetric complications (16%) (e.g., anemia, cardiovascular disorders), that occur during pregnancy, childbirth, or within 42 days after delivery [3, 4]. Deaths are often due to delays in seeking help, timely access to services, or poor quality of services, thereby highlighting the importance of monitoring maternal deaths and closing the gaps that lead to these deaths [5].

Improving maternal survival aligns with the Sustainable Development Goal 3: Ensure healthy lives and promote well-being for all ages (Target 3.1: Reduce the global maternal mortality ratio to less than 70 per 100,000 live births by 2030) [6]. To reduce maternal mortality, the Tanzanian Ministry of Health (MoH) and global partners supported the “Reducing Maternal Deaths in Tanzania” Program (hereafter referred to as the “Program”). The Program was implemented from 2006 to 2018 through a public-private partnership [7]. The Program resulted in a 43% reduction of institutional maternal mortality between 2013 and 2018 in the Kigoma region, a predominantly rural western region with the poorest maternal care indicators at the time of program initiation [7]. The Program was funded by Bloomberg Philanthropies, supported by the MoH and implemented by Thamini Uhai and EngenderHealth [7, 8]. The U.S. Centers for Disease Control and Prevention (CDC) supported the monitoring and evaluation of the Program [7]. The Program’s primary focus was on saving women and newborn lives through improved Emergency Obstetrics and Newborn Care (EmONC) services and contraceptive use; increased access to EmONC, including timely referrals; and increased quality of care in health facilities by providing equipment, supplies, and mentorship with supportive supervision and training of health workers to include training in Maternal Death Surveillance and Response (MDSR) implementation [9].

The MDSR is a system of identification, notification, review, and analyses of maternal deaths to identify medical and non-medical factors that contributed to a death and formulate actions to prevent future deaths [10]. Many countries, including Tanzania, have modified the MDSR process to include perinatal deaths [11]. In Tanzania, perinatal deaths include fetal deaths occurring at or after 28 weeks of gestation and neonatal deaths occurring during the first week after birth [11]. Although maternal and perinatal death reviews are generally well accepted [12] and may lead to mortality declines and improved quality of care when implemented with other interventions such as development of local leadership and training [13], there is little documentation on how widely the system is used and what facility characteristics are more likely to be associated with readiness for implementing Maternal and Perinatal Death Surveillance and Response (MPDSR) [13–15].

In Tanzania, implementation of MPDSR is often associated with systemic, contextual, and individual challenges [16]. Although the national MPDSR guidance emphasizes a culture of “no blame, no shame” [11], few MPDSR reviews adhere to this requirement, posing a significant barrier to successful reviews as some facilities may not ensure staff confidentiality, resulting in disciplinary action following review [17]. Knowledge levels of MPDSR among health providers vary by the facility level, geographical location, and staff exposure to MPDSR training [18]. The lack of implementation of recommendations from reviews aimed at improving practice may also be a barrier in some facilities [17]. Additionally, the absence of high-quality patient data can hinder the facilitation of these reviews [19].

Our study assessed the level of MPDSR implementation readiness in hospitals and health centers in the Kigoma region, the phase of MPDSR implementation in each facility, and the relationship between facility characteristics and MPDSR implementation. We also paid particular attention to those factors that may influence sustainable MPDSR practice. Despite some knowledge about the phases of implementation of MPDSR in health facilities in certain regions in Tanzania [17–19], the readiness of each health facility to implement and maintain MPDSR according to national guidelines and facility characteristics has not been previously explored. To the best of our knowledge, this study is the first to examine associations between sustainable MPDSR practice and health facility characteristics. Better understanding of facility readiness for MPDSR may help regional and national managers to focus implementation efforts to further reduce preventable maternal mortality.

## Methods

### Study setting

Situated in the western zone of Tanzania and bordering Lake Tanganyika, Burundi and Democratic Republic of Congo, Kigoma is a largely rural (82%) region with an area of 45,066 square kilometers and a population of 2,470,967 in 2022 [20]. Economic activity revolves around agriculture in 3 of the 4 districts (Kigoma rural, Kasulu, and Kibondo), while trade and fishing are the main activities in Kigoma urban [21]. Transportation challenges, while common in other regions because of low population density and poor road infrastructure, are exacerbated in Kigoma where only 2 major roads are paved. Some roads along the lake are impassable for most of the year, and poor road conditions prevent one third of the population access to emergency obstetric services within 2 h even during the dry season [21, 22]. Delivery care services are provided in dispensaries (primary health care units that provide skilled birth care largely for uncomplicated deliveries), health centers and hospitals. In 2022, there were 162 dispensaries, 29 health centers and 11 hospitals providing delivery care in the region. The majority (93%) of facilities providing maternity care were government owned [23]. Use of childbirth health services was low at the beginning of the Program (33% of women delivered in a health facility in 2010) [24] but increased substantially during the Program (from 47% in 2014 to 85% in 2018) [25], and continued to increase post-program (98% in 2022) [23].

### Study design

A cross-sectional study design was used to assess MPDSR implementation readiness in Kigoma health facilities. Our study was nested within a mixed-methods post-Program sustainability evaluation, funded by Bloomberg Philanthropies and executed by the national and regional health authorities in Kigoma with technical support from CDC.

### Sampling

The study included data collection from all public and private health facilities that conducted at least 90 deliveries per year. Data were collected during February-May 2023 from 11 hospitals, 29 health centers and 162 dispensaries. The evaluation activities were modelled after the evaluation methods and approaches that had been used to assess the Program’s impact during 2013–2018 [22, 26], and aimed to assess post-Program capacity and functionality of maternal care services and pregnancy outcomes.

### Data collection

Data collection consisted of a Health Facility Assessment (HFA) to gather information on maternity care infrastructure, human resources, and adherence to safe motherhood protocols and practices, drugs, equipment, and supplies. The HFA was based off the EmONC toolkit developed by Columbia University’s Mailman School of Public Health Averting Maternal Death and Disability group [27]. The HFA was also used to characterize the facility EmONC status, assess capacity and use of transport for emergency referrals, and examine facility readiness to conduct MPDSR [10]. Additionally, data collection included a Pregnancy Outcomes Monitoring Study (POMS) that was conducted at the same time as the HFA. The POMS methodology, developed by the CDC in 2013, included a system and tools for periodic data collection of all births occurring in health facilities providing maternity care in the region [23, 26]. The methodology consists of retrospectively collected individual data on all facility deliveries, including maternal characteristics, pregnancy outcomes, delivery details, obstetric complications, and obstetric and newborn interventions that occurred in facilities during the evaluation period [23].

CDC provided technical assistance to the HFA and POMS on questionnaire development, data collection, data analysis, and report writing [23, 28]. Questions about MPDSR included in the HFA questionnaire were adapted from previous MPDSR evaluations conducted in Tanzania [17, 19].

Data on MPDSR in each facility were obtained by interviewing the MPDSR facility focal point who was responsible for overseeing and maintaining the MPDSR process in the facility. Data collected from the MPDSR module administered in all hospitals (*n* = 11) and all health centers (*n* = 29) were combined with examination of data sources that attest to MPDSR practice in each facility (i.e., MPDSR death notification forms, MPDSR review forms, narrative/clinical summaries, and action plans). HFA data from hospitals and health centers were also combined with data collected at the same time through POMS.

### Measures

#### Outcome variable: facility MPDSR implementation readiness

**Fig. 1:**
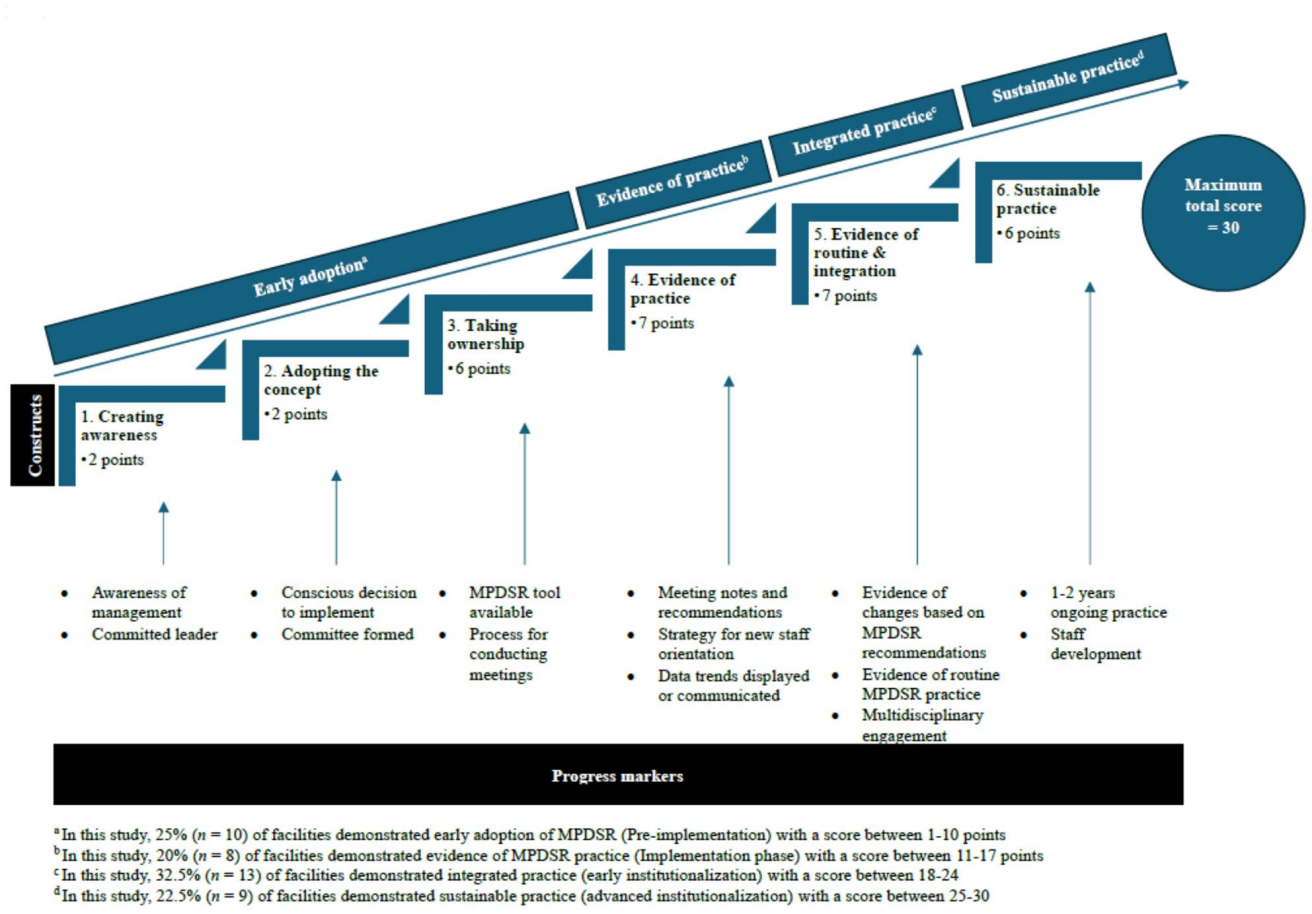
Phases of MPDSR implementation in health facilities by implementation constructs and progress markers: Kigoma, 2023

We used the results of the HFA to assess facility readiness to implement and maintain MPDSR processes based on attributes considered essential to MPDSR [17, 19, 29]. Using the presence or absence of these attributes in each facility and a standardized scoring system with progress markers, previous studies defined 6 constructs of implementation readiness: creating awareness (2 points), adopting the concept (2 points), taking ownership (6 points), evidence of practice (7 points), evidence of routine and integration (7 points), and evidence of sustainable practice (6 points) (Additional File 1) [17, 19, 29]. Applying the same approach, we assigned a score from 0 to 30 to all hospitals and health centers in the Kigoma region according to their construct-specific implementation attributes. The implementation readiness phase of each facility was based on the cumulative score of MPDSR attributes, presence of progress markers for each construct, and construct thresholds defined in the literature [17, 19, 29]. Progress markers are deemed as essential attributes that must be present in a facility to allow it to move to the next MPDSR implementation phase. Because most facilities in the region have already shown early evidence of MPDSR practice (i.e., had scores over 10 points), we considered facilities whose scores and progress markers corresponded to the first 3 constructs as being in an “early adoption” phase. Therefore, we present the distribution of health facilities by 4 phases of implementation readiness: early adoption (creating awareness, adopting the concept, and taking ownership), 1–10 points; evidence of practice, 11–17 points; integrated practice (early stage of institutionalization), 18–24 points; and sustainable practice (advance stage of institutionalization), 25–30 points (Fig. 1: Phases of MPDSR implementation in health facilities by implementation constructs and progress markers: Kigoma, 2023).

#### Independent variables: facility characteristics

The following facility characteristics derived from the HFA and POMS were used in our analyses: facility type (hospital or health center), facility ownership (governmental or private), facility location (urban or rural), sufficiency of personnel involved in delivery care in the facility (assessed by comparing currently occupied positions with designated positions, per national guidelines) [30], EmONC services—defined as performance of all 7 basic EmONC (BEmONC) or 9 comprehensive EmONC (CEmONC) life-saving interventions (i.e., signal functions) in the 3 months prior to the HFA, facility delivery volume (i.e., the number of facility deliveries assisted in 2022), facility volume of obstetric complications (i.e., direct obstetric complications treated as a proportion of all deliveries in 2022), proportion of delivery personnel trained in EmONC in the last 12 months, and proportion of facilities with personnel who received in-service MPDSR training over a duration of 1–3 days on the national MPDSR guidelines, processes and tools. Facility location was based on the Tanzania Census tracts [20].

We present descriptive analyses of MPDSR attributes and the distribution of facility characteristics by their implementation readiness phase as numbers and percentages. We used Fisher’s Exact test to test the association between facility characteristics and evidence of sustainable MPDSR practice. For all analyses, *alpha* < 0.05 was used for determining statistical significance. Analyses were performed using SAS version 9.4.

### Ethics statement

The sustainability evaluation was approved by the National Institute for Medical Research (NIMR) in Tanzania as part of a larger set of activities to support the Tanzania MoH assessment of the Program sustainability in Kigoma. The sustainability evaluation activities were determined to be non-research, public health practice by the Centers for Disease Control and Prevention. Written consent from the focal point was obtained prior to the interview and documentation examination, in accordance with ethics committee approval. NIMR approval was used to obtain consent from the Facility In-charge or the Facility Director prior to collecting routine health facility data (NIMR approval #: NIMR/HQ/R.8c/Yol.l/2077, issued on June 28, 2022).

## Results

### MPDSR attributes

The attributes for each of the 6 constructs of implementation readiness are reported in Table 1 by facility type (11 hospitals and 29 health centers).

**Table 1:** MPDSR constructs and attributes in health facilities by facility type: Kigoma, 2023

MPDSR constructs/attributes^a^		Facility Type	
	Total *N*= 40 (%)	Hospital *n* = 11 (%)	Health Center *n* = 29 (%)
**Created awareness**			
There is a Maternal and Perinatal Death Surveillance and Response (MPDSR) coordinator at the facility.	95.0%	100%	93.1%
The facility received support from key leaders or change agents (District Health Manager/CEO/Superintendent or Facility Director) in the MPDSR implementation.	87.5%	90.9%	86.2%
**Adopted the system**			
The facility has a formal system for reviewing maternal deaths, stillbirths, neonatal deaths or near-misses.	95.0%	100%	93.1%
The facility has an MPDSR review committee.	95.0%	100%	93.1%
**Took ownership**			
Standard maternal and perinatal mortality audit forms are available.	90.0%	90.9%	89.7%
There is a written documentation system for tracking the follow-up on specific recommendations.	62.5%	72.7%	58.6%
Ability to show documentation of meetings process.	72.5%	90.9%	65.5%
Code of conduct agreement available.	92.5%	100%	89.7%
In the past year, the facility received support (financial or in-kind) from the facility or district budget or from another partner to support the implementation of maternal/perinatal death review.	52.5%	45.5%	55.2%
**Evidence of practice**			
Meeting minutes are taken during MPDSR reviews.	87.5%	100.0%	82.8%
An action plan developed as part of the review process.	72.5%	100.0%	62.1%
Meeting minutes include follow-up from previous meetings.	47.5%	54.6%	44.8%
Confidentiality of staff and patients maintained	70.0%	100.0%	58.6%
MPDSR educational activities were conducted in the past year for facility staff (on-site or off-site) and a copy of the National MPDSR guidance issued by the Ministry of Health is available.	85.0%	90.9%	82.8%
There are statistics or data trends related to MPDSR displayed somewhere in the facility.	95.0%	100.0%	93.1%
**Evidence of routine & integration**			
There is evidence that recommendations made during the mortality audit process resulted in a change in how care was provided.	82.5%	90.9%	79.3%
Frequency of audit meetings			
Frequent (Within a week following a maternal/perinatal death)	0.0%	0.0%	0.0%
Moderately frequent (Monthly or bi-monthly or quarterly or every 6 months)	95.0%	100.0%	93.1%
Not frequent (Less than twice per year or only when budget and personnel are available)	5.0%	0.0%	6.9%
Death review meetings include staff from different disciplines, management.	72.5%	90.9%	65.5%
Recommendations from facility-based death reviews are fed back to the community (e.g., Health Board Meetings, via Community Health Workers, or through meetings with community leaders).	65.0%	63.6%	65.5%
**Evidence of sustainable practice**			
MDSR and PDSR started in 2022 or earlier	92.5%	90.9%	93.1%
There are educational activities to introduce MPDSR to staff members	87.5%	90.9%	86.2%
The facility has trained staff in MPDSR	57.5%	63.6%	55.2%

^a^Constructs and attributes adapted from previous studies [17, 19, 29]

Created awareness **-** All hospitals (100%) and 93.1% of health centers reported having a MPDSR coordinator. Most hospitals (90.9%) and health centers (86.2%) reported receiving support from key leaders or change agents (i.e., District Health Manager/CEO/Superintendent or Facility Director) in implementing the MPDSR system.

Adopted the system **–** Most facilities (100% of hospitals and 93.1% of health centers) reported having a formal system and committees for reviewing maternal deaths, stillbirths, neonatal deaths or near-misses.

Took ownership **-** Most facilities (90.9% of hospitals and 89.7% of health centers) reported having standard maternal mortality and perinatal audit forms available. More hospitals (72.7%) compared to health centers (58.6%) reported having a written documentation system for tracking the follow-up on specific recommendations. Fewer health centers (65.5%) than hospitals (90.9%) were able to show documentation of the review process (i.e., image of action plan, assignment of individuals to follow-up on an action plan, meeting attendance list, a completed maternal or perinatal death review, presence of MoH MPDSR guidance). All hospitals (100%) and 89.7% of health centers had a code of conduct agreement available in the reviews. About half of the facilities (45.5% of hospitals and 55.2% of health centers) reported receiving financial or in-kind support from the facility or district budgets or from another partner to support MPDSR implementation.

Evidence of practice **-** All hospitals (100%) and 82.8% of health centers reported having meeting minutes recorded during MPDSR reviews. All hospitals (100%) reported having an action plan developed as part of the review process, compared to 62.1% of health centers; however, only half of the facilities (54.6% of hospitals and 44.8% of health centers) followed-up on recommendations from previous meetings in their meeting minutes. All hospitals (100%) but only 58.6% of health centers provided evidence of maintaining confidentiality of staff and patients during reviews. Most facilities (90.9% of hospitals and 82.8% of health centers) reported having MPDSR educational activities in the past year for staff and a copy of the National MPDSR guidance issued by the MoH available. All hospitals (100%) and 93.1% of health centers reported displaying statistics or data trends related to MPDSR in the facility.

Evidence of routine and integration **–** Most facilities (90.9% of hospitals and 79.3% of health centers) provided evidence that recommendations made during the review process resulted in concrete changes. All hospitals (100%) and 93.1% of health centers reported having audit meetings monthly, bi-monthly, quarterly or at least every other quarter, depending on the number of deaths needing to be reviewed; however, two health centers (6.9%) reported having infrequent audit meetings (less than twice per year or only when budget and personnel were available). Most hospitals (90.9%) and 65.5% of health centers reported that death review meetings included staff across different disciplines. Only 63.6% of hospitals and 65.5% of health centers reported that recommendations from facility-based death reviews were communicated to the communities where the decedents resided.

Evidence of sustainable practice **-** Most facilities (90.9% of hospitals and 93.1% of health centers) reported that MPDSR started at the facility in 2022 or earlier; and 90.9% of hospitals and 86.2% of health centers conducted educational activities to introduce MPDSR to staff members. Fewer facilities (63.6% of hospitals and 55.2% of health centers) reported staff were trained in MPDSR.

### MPDSR implementation readiness phases

Based on the facility attributes and progress markers used to determine MPDSR implementation readiness phases, 10 facilities (25%) were classified as being in the early adoption phase, 8 (20%) in the evidence of practice phase, 13 (32.5%) in the integrated practice phase, and 9 (22.5%) in the sustainable practice phase (Fig. 1; Table 2).

**Table 2:** A descriptive analysis of the relationship between facility characteristics and MPDSR implementation readiness phases: Kigoma health facility assessment, 2023

Facility Characteristic	Total (40) *N* (%)	MPDSR implementation readiness phases			
		Early Adoption^a^ Total = 10 *n *(%)	Evidence of Practice^b^ Total = 8 *n* (%)	Integrated Practice^c^ Total = 13 *n *(%)	Sustainable Practice^d^ Total = 9 *n *(%)
Facility type					
Hospital	11 (27.5)	2 (20.0)	0 (0.0)	4 (30.8)	5 (55.6)
Health Center	29 (72.5)	8 (80.0)	8 (100.0)	9 (69.2)	4 (44.4)
Facility ownership					
Government	33 (82.5)	9 (90.0)	7 (87.5)	11 (84.6)	6 (66.7)
Private/faith-based	7 (17.5)	1 (10.0)	1 (12.5)	2 ( 15.4)	3 (33.3)
Facility location					
Urban	9 (22.5)	1 (10.0)	0 (0.0)	2 (15.4)	6 (66.7)
Rural	31 (77.5)	9 (90.0)	8 (100.0)	11 (84.6)	3 (33.3)
Staffing gap^e^					
Clinicians					
No staffing gap	21 (52.5)	4 (40.0)	3 (37.5)	8 (61.5)	6 (66.7)
Yes, there is a staffing gap	19 (47.5)	6 (60.0)	5 (62.5)	5 (38.5)	3 (33.3)
Clinical Officer/Assistant					
No staffing gap	31 (77.5)	7 (70.0)	6 (75.0)	10 (76.9)	8 (88.9)
Yes, there is a staffing gap	9 (22.5)	3 (30.0)	2 (25.0)	3 (23.1)	1 (11.1)
Midwife/nurse officer/enrolled nurse					
No staffing gap	19 (47.5)	2 (20.0)	3 (37.5)	6 (46.2)	8 (88.9)
Yes, there is a staffing gap	21 (52.5)	8 (80.0)	5 (62.5)	7 (53.9)	1 (11.1)
EmONC services^f^					
Facility with EmONC services	26 (65.0)	5 (50.0)	4 (50.0)	10 (76.9)	7 (77.8)
Facility without EmONC services	14 (35.0)	5 (50.0)	4 (50.0)	3 (23.1)	2 (22.2)
Facility volume^g^					
Low (< 1000)	21 (52.5)	7 (70.0)	5 (62.5)	7 (53.9)	2 (22.2)
Medium (1000–2999)	16 (40.0)	3 (30.0)	3 (37.5)	5 (38.5)	5 (55.6)
High (≥ 3000)	3 (7.5)	0 (0.0)	0 (0.0)	1 (7.7)	2 (22.2)
Proportion of deliveries with direct obstetric complications^h^					
Low (< 10%)	22 (55.0)	6 (60.0)	6 (75.0)	9 (69.2)	1 (11.1)
Medium (10% ≤ x < 30%)	15 (37.5)	4 (40.0)	2 (25.0)	3 (23.1)	6 (66.7)
High (≥ 30%)	3 (7.5)	0 (0.0)	0 (0.0)	1 (7.7)	2 (22.2)
Proportion of delivery personnel trained in EmONC in the last 12 month^i^					
<60%	9 (22.5)	4 (40.0)	2 (25.0)	2 (15.4)	1 (11.1)
≥60%	31 (77.5)	6 (60.0)	6 (75.0)	11 (84.6)	8 (88.9)
Facilities have personnel trained in MPDSR					
Yes	23 (57.5)	6 (60.0)	4 (50.0)	4 (30.8)	9 (100.0)
No	17 (42.5)	4 (40.0)	4 (50.0)	9 (69.2)	0 (0.0)

*Abbreviations*: *EmONC* emergency obstetric and newborn care, *MPDSR* Maternal and Perinatal Death Surveillance and Response

Percentages may not sum up to 100 due to rounding

^a^Early adoption (Pre-Implementation phase score) (1-10 points)

^b^Evidence of practice (Implementation phase score) (11-17 points)

^c^Integrated practice phase score (Early institutionalization) (18-24 points)

^d^Sustainable practice phase score (Advanced institutionalization) (25-30 points)

^e^Staffing gap for clinicians (Obstetricians/gynecologists, general surgeons, medical doctors, assistant medical officers, anesthetists) was calculated by subtracting the number of clinicians in the facility providing maternal and newborn health from the minimum number of designated clinician positions in the facility. The number of designated clinician positions in a facility was calculated by summing up corresponding clinician positions according to the type of facility (hospital or health center). Staffing gap for clinical officers/assistants engaged was calculated by subtracting the number of clinical officers/assistants in the facility providing maternal and newborn health from the minimum number of designated clinical officers/assistants in the facility. Similarly, staffing gap for midwives, nurse officers and enrolled nurses was calculated by subtracting the number of these cadre in the facility from the minimum number of designated cadre positions. The number of designated midwives, nurse officers and enrolled nurses was calculated by summing up these cadre positions according to the type of facility. If the result of the subtraction was greater than 0, the facility had a staffing gap for that category of cadre

^f^Basic EmONC interventions include administration of parenteral antibiotics, uterotonics, or anticonvulsants; manual removal of placenta; removal of retained products; assisted vaginal delivery; and basic neonatal resuscitation. Comprehensive EmONC interventions include two additional services: ability to perform obstetric surgery (e.g., C- section) and blood transfusion. Facilities were classified based on whether they had, within the previous 3 months, performed each of these interventions. Because assisted vaginal delivery—using either forceps or vacuum extractor—is relatively uncommon in Tanzania, some facilities were classified as fully providing EmONC care even if they did not perform assisted vaginal deliveries within the past 3 months.

^g^Number of deliveries in the facility during a calendar year (year 2022 is used to estimate the facility delivery volume)

^h^Proportion of deliveries in 2022 with direct obstetric complications that include antepartum and postpartum hemorrhage, uterine rupture, prolonged or obstructed labor, postpartum sepsis, eclampsia/preeclampsia; excluding care for first trimester complications (complicated abortions and ectopic pregnancies)

^i^Proportion of hospital and health center staff receiving EmONC training in 2022/2023

### A descriptive analysis of the relationship between facility characteristics and MPDSR implementation readiness phases

Among facilities in the sustainable practice phase (*n* = 9), more facilities were hospitals (55.6%) than health centers (44.4%) (Table 2). Among sustainable practice facilities, two-thirds (66.7%) were government facilities and 33.3% were private/faith-based facilities. For all phases except sustainable practice, most facilities were located in rural areas (77.5%). Most (66.7%) urban facilities demonstrated sustainable practice. Very few facilities in the sustainable phase had a gap in practicing clinicians (33.3%), clinical officers (11.1%) and nurses (11.1%).

Most facilities (77.8%) that demonstrated sustainable practice were EmONC facilities. Most facilities demonstrating sustainable practice had a medium (55.6%) or high (22.2%) volume of deliveries per year, while facilities in earlier MPDSR implementation phases had lower delivery volumes. Among facilities that demonstrated sustainable practice, most had a medium (66.7%) or high (22.2%) volume of women with direct obstetric complications as a proportion of their total deliveries per year, while 11.1% had a low volume. Most facilities (88.9%) that demonstrated sustainable practice also reported having ≥ 60% of trained personnel in EmONC in the last 12 months. Further, all (100%) facilities in the sustainable practice phase reported that they employed personnel trained in MPDSR.

Sustainable MPDSR practice was significantly associated with an urban facility location (*p* = 0.0014), not having a staffing gap for midwives and nurses (*p* = 0.0072), having a high facility delivery volume (*p* = 0.0472), having high volume of obstetric complications treated (*p* = 0.0036), and having personnel trained in MPDSR in the facility (*p* = 0.0051; Table 3).

**Table 3:** Associations between facility characteristics and sustainable MPDSR practice: Kigoma Region, 2023

Facility Characteristics	Total (40) *N *(%)	Sustainable Practice^a^ Total = 9 *n *(%)	Not Sustainable Practice Total = 31 *n *(%)	*p* value^b^
Facility type				0.0834
Hospital	11 (27.5)	5 (55.6)	6 (19.4)	
Health Center	29 (72.5)	4 (44.4)	25 (80.6)	
Facility ownership				0.3165
Government	33 (82.5)	6 (66.7)	27 (87.1)	
Private/faith-based	7 (17.5)	3 (33.3)	4 (12.9)	
Facility location				**0.0014**
Urban	9 (22.5)	6 (66.7)	3 (9.7)	
Rural	31 (77.5)	3 (33.3)	28 (90.3)	
Staffing gap^c^				
Clinicians				0.4570
No staffing gap	21 (52.5)	6 (66.7)	15 (48.4)	
Yes, there is a staffing gap	19 (47.5)	3 (33.3)	16 (51.6)	
Clinical Officer/Assistant				0.6538
No staffing gap	31 (77.5)	8 (88.9)	23 (74.2)	
Yes, there is a staffing gap	9 (22.5)	1 (11.1)	8 (25.8)	
Midwife/nurse officer/enrolled nurse				**0.0072**
No staffing gap	19 (47.5)	8 (88.9)	11 (35.5)	
Yes, there is a staffing gap	21 (52.5)	1 (11.1)	20 (64.5)	
EmONC services^d^				0.4527
Facility with EmONC services	26 (65.0)	7 (77.8)	19 (61.3)	
Facility without EmONC services	14 (35.0)	2 (22.2)	12 (38.7)	
Facility volume^e^				**0.0472**
Low (< 1000)	21 (52.5)	2 (22.2)	19 (61.3)	
Medium (1000–2999)	16 (40.0)	5 (55.6)	11 (35.5)	
High (≥ 3000)	3 (7.5)	2 (22.2)	1 (3.2)	
Proportion of deliveries with direct obstetric complications^f^				**0.0036**
Low (< 10%)	22 (55.0)	1 (11.1)	21 (67.7)	
Medium (10% ≤ x < 30%)	15 (37.5)	6 (66.7)	9 (29.0)	
High (≥ 30%)	3 (7.5)	2 (22.2)	1 (3.2)	
Proportion of delivery personnel trained in EmONC in the last 12 months^g^				0.6538
<60%	9 (22.5)	1 (11.1)	8 (25.8)	
≥60%	31 (77.5)	8 (88.9)	23 (74.2)	
Facilities have personnel trained in MPDSR				**0.0051**
Yes	23 (57.5)	9 (100.0)	14 (45.2)	
No	17 (42.5)	0 (0.0)	17 (54.8)	

*Abbreviations*: *EmONC* emergency obstetric and newborn care, *MPDSR* Maternal and Perinatal Death Surveillance and Response

Percentages may not sum up to 100 due to rounding

^a^Sustainable practice phase score (25-30 points)

^b^Fisher’s Exact test was used to test for associations between facility characteristics and facilities with sustainable MPDSR practice. All significant p values are bolded

^c^Staffing gap for clinicians (Obstetricians/gynecologists, general surgeons, medical doctors, assistant medical officers, anesthetists) was calculated by subtracting the number of clinicians in the facility providing maternal and newborn health from the minimum number of designated clinician positions in the facility. The number of designated clinician positions in a facility was calculated by summing up corresponding clinician positions according to the type of facility (hospital or health center). Staffing gap for clinical officers/assistants engaged was calculated by subtracting the number of clinical officers/assistants in the facility providing maternal and newborn health from the minimum number of designated clinical officers/assistants in the facility. Similarly, staffing gap for midwives, nurse officers and enrolled nurses was calculated by subtracting the number of these cadre in the facility from the minimum number of designated cadre positions. The number of designated midwives, nurse officers and enrolled nurses was calculated by summing up these cadre positions according to the type of facility. If the result of the subtraction was greater than 0, the facility had a staffing gap for that category of cadre.

^d^Basic EmONC interventions include administration of parenteral antibiotics, uterotonics, or anticonvulsants; manual removal of placenta; removal of retained products; assisted vaginal delivery; and basic neonatal resuscitation. Comprehensive EmONC interventions include two additional services: ability to perform obstetric surgery (e.g., C- section) and blood transfusion. Facilities were classified based on whether they had, within the previous 3 months, performed each of these interventions. Because assisted vaginal delivery—using either forceps or vacuum extractor—is relatively uncommon in Tanzania, some facilities were classified as fully providing EmONC care even if they did not perform assisted vaginal deliveries within the past 3 months.

^e^Number of deliveries in the facility during a calendar year (year 2022 is used to estimate the facility delivery volume)

^f^Proportion of deliveries with complications that include antepartum and postpartum hemorrhage, uterine rupture, prolonged or obstructed labor, postpartum sepsis, eclampsia/preeclampsia; excluding first trimester complications (excluding complicated abortions and ectopic pregnancies)

^g^Proportion of hospital and health center staff receiving EmONC training in 2022/2023

## Discussion

We assessed MPDSR implementation in all hospitals and health centers (*n* = 40) in the Kigoma region using facility attributes related to MPDSR practice. Based on the facility attributes and progress markers, we classified facilities into implementation readiness phases according to the literature [17, 19, 29]. We found that facilities in this study were spread across the continuum of MPDSR implementation, with only 9 facilities (22.5%) reaching sustainable implementation and 13 (32.5%) demonstrating integrated practice.

A study conducted in Mara and Kagera regions in Tanzania in 2017 among 16 selected hospitals and health centers reported that only 1 hospital (6.3%) had reached sustainable MPDSR practice, while 56.3% of facilities showed evidence of integrated practice [17]. Similar findings were also reported by a study conducted in 2016–2017 in 55 selected health facilities from 4 sub-Saharan African countries, including Tanzania; only 11% of facilities assessed by this study were classified as having sustainable MPDSR practice and 55% had evidence of integrated practice [29]. In contrast, a study of 38 hospitals and health centers (out of 67 in the region) conducted in the Morogoro region, Tanzania in 2020 found that only 8% of facilities had integrated practice and zero had sustainable practice, which highlights the difference in MPDSR implementation [19]. While findings are not strictly comparable because we assessed all hospitals and health centers in the Kigoma region and not just a purposefully selected sample, only 55% of hospitals and health centers in this study in 2023 showed integrated or sustainable practice, suggesting gaps in MPDSR implementation.

Information is limited on the association between facility location and sustainable practice. In this study, sustainable MPDSR practice was associated with urban facility location, while facilities in rural areas were often classified as being in early stages of MPDSR implementation. An earlier study of the Program in Kigoma demonstrated that it was possible to improve maternal and reproductive health in remote low-resource settings through a combination of factors such as long-term investment, active multi-partner collaboration with the government as a central partner, appropriate context-driven interventions, well-functioning and high-quality clinical services, and responsiveness to evaluation data [7].

Sustainability in MPDSR practice was also significantly associated with adequate staffing for midwives and nurses. Currently, the shortage for this cadre in Kigoma is higher than at the Program ending (the shortage increased from 3% in 2019 to 16% in 2023) while the shortage in medical doctors has disappeared (from 20% in 2019 to a small surplus in 2023) [28]. Considering that more midwives and nurses are necessary to ensure adequate functionality in maternities, including adequate MPDSR implementation, urgent recruitment of these cadre may be necessary to achieve facility MPDSR sustainability. Although the Program contributed to improving the number of health providers in the region between 2013 and 2018 by 64%, the density of skilled birth attendants remains nearly 7 times lower than the recommended minimum threshold (44.5 per 10,000 population) [26].

We suspect that facilities with no staffing gap are also more equipped to handle higher delivery volume and more complicated deliveries, giving them the bandwidth to achieve sustainable MPDSR practice. This may also explain the significant associations between sustainable MPDSR practice and facility volume, including the volume of complicated deliveries treated. Caseload, while it may place a burden on health providers, is an important determinant in maintaining skills and clinical proficiency. Lack of practical opportunities due to low caseloads could lead to a significant reduction of knowledge and skills over time. Large volume facilities provide learning opportunities not just in maintaining clinical skills but also in institutionalization of MPDSR practice. Health facilities and providers can make better informed decisions about how to improve the quality of care they deliver before, during and after childbirth when they review complex cases and learn from an array of direct and contributing factors that may have led to a death. Low-volume facilities are more likely to have staffing gaps and to have fewer opportunities to institutionalize MPDSR. Other healthcare system constraints such as the lack of sufficient medical recording systems (e.g., a computer server) and the lack of mechanisms for keeping patients’ information may also be more pronounced in these facilities [31]. The fear of blame and lack of motivation among staff may also impact how they view and implement MPDSR [32]. The principles of “No Name, No Blame, and No Shame,” are meant to enable a full understanding of the chain of events leading to a death, allowing for the identification of gaps and best solutions; however, this is often not the prevailing culture given the hierarchical structures and limited human resources in facilities, affecting opportunity for honest dialogue and assumption of responsibility [11, 33].

Successful implementation of MPDSR is also dependent on the availability of staff from multiple disciplines that are required to attend review meetings, as stipulated by MoH guidance [17]. Staff who have been trained in MPDSR are more likely to have positive perceptions towards implementing the system, which may explain the significant relationship between having facility personnel trained in MPDSR and sustainable practice. Similar findings were reported in another study in Morogoro region, Tanzania, where they recommended the training of facility personnel so as to increase their positive attitude and use of the MPDSR system [34]. Greater accountability among facility administrators may affect the MPDSR training availability for facility personnel and their buy-in of the MPDSR system.

An important consideration is that the number of hospitals and health centers in Kigoma increased from 33 to 40 between 2019 and 2023 including the addition of 5 new hospitals and upgrades of dispensaries to become health centers [28]. These newly established or upgraded facilities may encounter challenges in achieving sustainable MPDSR practices due to limited staffing capacity, inadequate infrastructure and equipment, as well as issues with communication and transport. Furthermore, these newer facilities have had fewer years of experience with MPDSR practice and may not yet have adopted the MPDSR.

Another important barrier noted in a previous study is the inability of facility staff to implement the action plans developed due to lack of resources; as a result, the same mistakes are repeated [16]. Most action plans that we reviewed were often not specific, measurable, attainable, relevant, or time bound, similar to findings from the Lindi and Mtwara regions, Tanzania [35]. Another study noted that, often, poor documentation resulting in inconsistencies and incompleteness led to unclear action plans and the inability to follow up on recommendations [31]. This study found that although action plans were developed in about 73% of all facilities, less than half reported follow-up activities related to previous meetings in their meeting minutes, making it difficult to ascertain what actions, if any, were taken, and to monitor potential improvements over time. Overall, the implementation of MPDSR in the Kigoma region will require additional effort at the facility level to ensure consistent implementation.

While this study leverages a comprehensive assessment of all 40 hospitals and health centers in the Kigoma region and may serve as an example for other studies, the results may not be directly applicable to other regions and/or healthcare settings. Another limitation is that the responses provided on the implementation of MPDSR may be influenced by social desirability bias. To mitigate the impact of potentially inaccurate self-reported data, authors gathered information from other sources, including a review of MPDSR documentation, which provides additional insight on the implementation process. A third limitation is that assessing the implementation of MPDSR based on existing methods does not fully address the quality of implementation [29]. For example, we did not assess whether causes of death were correctly assigned. In addition, the limited number of health facilities in the Kigoma region (*n* = 40) prevented us from assessing whether significant associations were confounded by other covariates. Finally, we also acknowledge that other efforts from the Tanzanian government and non-governmental organizations may have also contributed to facility and health service improvements in the region and may influence the findings.

## Conclusion

Achieving MPDSR sustainability in Kigoma has been slow. Facilities that have achieved MPDSR sustainability may be more prepared to handle delivery complications and a higher volume of deliveries when needed primarily because they have an adequate number of skilled personnel. Having trained personnel in MPDSR may also be important for the effective implementation of the MPDSR system and its long-term sustainability.

## Supplementary Information

Supplementary Material 1

## Data Availability

Data may be obtained from a third party and are available upon request. All data that support the findings of the sustainability evaluation are archived in the libraries of the CDC Foundation. While the data were not approved to be made publicly available by NIMR, there are standard procedures in place for other researchers to request the data that could be used with the permission of the Government of Tanzania. Requests for permission to access data can be submitted to NIMR to ensure these data are appropriate for the use sought, will be used consistent with any applicable legal restrictions on the data, and will be used for an appropriate purpose.

## Abbreviations

BEmONC: Basic Emergency Obstetrics and Newborn Care
CDC: Centers for Disease Control and Prevention
CEmONC: Comprehensive Emergency Obstetrics and Newborn Care
EmONC: Emergency Obstetrics and Newborn Care
HFA: Health Facility Assessment
MDSR: Maternal Death Surveillance and Response
MoH: Tanzania Ministry of Health
MPDSR: Maternal and Perinatal Death Surveillance and Response
NIMR: National Institute for Medical Research
POMS: Pregnancy Outcomes Monitoring Study
