# Supplementary material for: Assessment of maternal and perinatal death surveillance and response (MPDSR) implementation in health facilities in Kigoma, Tanzania: a descriptive cross-sectional study
Source: BMC Pregnancy Childbirth. 2026 Feb 12;26:335. doi: 10.1186/s12884-026-08775-1 (PMC13020112; doi:10.1186/s12884-026-08775-1)
Supplement: Supplementary file 1 — Supplementary Material 1 [file 12884_2026_8775_MOESM1_ESM.docx]

**Additional File 1.** Scoring for MPDSR implementation readiness constructs and corresponding progress markers and attributes ^a^

| **MPDSR readiness constructs** | **Progress markers** | **Attributes** |
| --- | --- | --- |
| 1. Created awareness (2 points) | Awareness by management | Is there a Maternal and Perinatal Death Surveillance and Response (MPDSR) coordinator at the facility?^b^ (1 point) |
|  | Committed leader | Did your facility receive support from any of the following people in the MPDSR implementation?^c^ (1 point) |
| 2. Adopted the system (2 points) | Conscious decision to implement | Does the facility have a formal system for reviewing maternal deaths, stillbirths, neonatal deaths or near-misses?^d^ (1 point) |
|  | Committee formed | Does the facility have a review committee for MPDSR?^e^ (1 point) |
| 3. Took ownership of the system (6 points) | MPDSR tools available | Are standard mortality audit forms used to collect and summarize details about the maternal and/or perinatal deaths that are reviewed?^f^ (1 point) |
|  |  | What system is used to classify cause of death on the mortality review forms?^f^ (1 point) |
|  |  | What system is used to classify non-medical factors that may have contributed to a maternal/perinatal death?^f^ (1 point) |
|  |  | Is there written documentation system for tracking the follow-up on specific recommendations?^g^ (1 point) |
|  | Process for conducting meetings | Is there documentation to describe or show meetings process?^h^ (1 point) |
|  |  | In the past year, has your facility received support (financial or in kind) from the hospital or district budget or from another partner to support the implementation of maternal/perinatal death reviews?^i^ (1 point) |
| 4. Evidence of practice (7 points) | Meeting notes and recommendations | Are meeting minutes taken?^j^ (1 point) |
|  |  | Do the minutes include follow-up from previous meetings?^k^ (1 point) |
|  |  | Is an action plan developed as part of the review process?^l^ (1 point)  Is a Clinical Summary prepared for each case prior to the meeting?^m^ (1 point) |
|  | Strategy for new staff orientation | Do you have a copy of the National MPDSR guidance issued by the Ministry of Health and Were any MPDSR education activities conducted in the past year for facility staff (on-site or off-site)?^n^ *(Proxy for face-to-face or written orientation to death reviews)* (1 point) |
|  | Data trends displayed or communicated | Are there any statistics or data trends related to MPDSR displayed somewhere in the facility?^o^ (2 points) |
| 5. Evidence of routine & integration (7 points) | Evidence of changes based on MPDSR recommendations | Recommendations made during the mortality audit process resulted in a change in how care was provided^p^ (3 points) |
|  | Evidence of routine MPDSR practice | Frequency of mortality audit meetings.^q^ (0.5 points) |
|  | Multidisciplinary engagement | Members of your review committee include staff from different disciplines and management.^r^ (2 points) |
|  |  | Are recommendations from facility-based death reviews fed back to the community in any way (e.g., Health Board Meetings, via Community Health Workers, meetings with community leaders)?^s^(1.5 points) |
| 6. Sustainability of MPDSR practice (6 points) | 1-2 years ongoing practice | Facility records show ongoing death review meetings for at least 1 year.^t^ (2 points) |
|  | Staff development | Do you have any educational activities in your facility to introduce MPDSR to staff members?^u^ (1 point) |
|  |  | This facility has trained personnel in MPDSR?^v^ (1 point) |
|  |  | Evidence of stages 1-5 completed (2 points) |
| **Maximum Total Score** |  | **30 points** |

^a^ Attributes used are from the MPDSR module of the 2023 Health Facility Assessment and information gathered through review of MPDSR documentation.

^b^ Respondents were asked, “Is there a Maternal Death Surveillance and Response (MDSR) coordinator at the facility?” AND “Is there a Perinatal Death Surveillance and Response (PDSR) coordinator at the facility?” Response choices were categorized as Yes (“Yes” OR “Yes, same as for MDSR” OR “Yes, separate from the MDSR coordinator”); No.

^c^ Respondents were asked, “Did your facility receive support from any of the following people in the MPDSR implementation?” Response choices for key support were categorized as Yes (“District Health Manager/CEO/Superintendent” OR “Facility Director”); No.

^d^ Respondents were asked, “Does the facility have a formal system for reviewing maternal deaths, still births, neonatal deaths or near-misses?” Response choices were categorized as Yes (“Yes, for maternal deaths” OR “Yes, for stillbirths” OR “Yes, for neonatal deaths” OR “Yes, for near-misses"); No.

^e^ Respondents were asked, “Does the facility have a review committee for MPDSR?” Response choices were categorized as Yes; No.

^f^ Respondents were asked if, “Standard maternal mortality audit forms (Maternal Death Review Form A),” AND “Standard perinatal mortality audit forms (Perinatal Death Review From A),” are available. Response choices were categorized as Yes; No. The same forms were used to assess system used to classify the cause of death and system used to classify non-medical factors.

^g^ Respondents were asked, “Is there a written documentation system for tracking the follow-up on specific recommendations?” Response choices were categorized as Yes; No.

^h^ The following documents in facilities were reviewed to show if facilities have an MPDSR meeting process in place: Image of action plan, assignment of individuals to follow-up on an action plan, meeting attendance list, a completed maternal or perinatal death review, presence of MoH MPDSR guidance. Only facilities that showed the presence of all these documents were considered as fully having an MPDSR meeting process in place.

^i^ Respondents were asked, “In the past year, has your facility received support (financial or in-kind) from the hospital or district budget or from another partner to support the implementation of maternal/perinatal death reviews?” Response choices were categorized as Yes (“Yes, received financial” OR “Yes, received in-kind" OR “Yes, both”); No.

^j^ Respondents were asked, “Are meeting minutes taken?” Response choices were categorized as Yes; No.

^k^  Respondents were asked, “Do the minutes include follow-up from previous meetings?” Response choices were categorized as Yes; No.

^l^ Respondents were asked, “Is an action plan developed as part of the review process?” Response choices were categorized as Yes; No.

^m^ Respondents were asked, “Is the Clinical Summary Form provided by the MoH or another form used to collect and summarize details about the maternal and/or perinatal deaths that are reviewed?” Response choices were categorized as Yes; No. This question was used as proxy for the maintenance of confidentiality.

^n^ Respondents were asked, “Were any MPDSR educational activities conducted in the past year for facility staff (onsite or off-site)?” AND “Do you have a copy of the National MPDSR guidance issued by the Ministry of Health?” Response choices were categorized as Yes (“Yes, on-site" OR “Yes, off-site" OR “Yes, both”); No. These questions were used a proxy for face-to-face orientation.

^o^ Respondents were asked, “Are there any statistics or data trends related to MPDSR displayed somewhere in the facility (e.g., poster or information sheet on a wall)?” Response choices were categorized as Yes; No.

^p^ Respondents were asked, “Can you tell us about a time where the recommendations made during the mortality audit process resulted in a change in how care was provided?” Response choices were categorized as Yes; No. Only those facilities that responded “Yes” and could provide concrete evidence of recommendations in the open-ended responses to the next question, “Summarize the example and type the response succinctly,” were included in the final “Yes” category.

^q^ Respondents were asked, “How frequently do mortality audit meetings take place in your facility?” Response choices were categorized as Frequent, Moderately Frequent, Not Frequent.

^r^ Respondents were asked, “Usually, who are the members of your review committee?” Only selected designations that included Chair AND Presenter AND Secretary AND Nurse in charge of labor ward AND Medical Officer at the Center AND Representative from Anesthetist AND Representative from Operating Theatre AND Nurse in charge of gynecological ward AND Hospital in-charge of laboratory services AND Hospital in-charge of Pharmacy AND Midwives were representative of a multi-disciplinary committee. This question was used as proxy for death review meetings include staff from different disciplines.

^s^ Respondents were asked, “Are recommendations from the facility-based death reviews fed back to the community in any way (e.g., Hospital Board meetings, via Community Health Workers, meetings with community leaders)?” Response choices were categorized as Yes; No.

^t^ Respondents were asked, “When was MDSR started at the facility?” or “When was PDSR started at the facility?” Only those responses with the year 2022 or earlier years showed evidence of MPDSR for more than 1 year.

^u^ Respondents were asked, “Do you have educational activities in your facility to introduce MPDSR to staff members” Response choices were categorized as Yes; No.

^v^ Respondents were asked if the facility has trained in MPDSR. Response choices were categorized as Yes; No.
